# How Is It Done?

**Published:** 1891-12-12

**Authors:** 


### Passing Topics.

### HOW IS IT DONE?

In these days of self-assertion it is more than ever true that people are ready to take a man at his own valuation. Modesty is no longer a virtue; in the struggle for existence it has become an imbecility. There may be some old-fashioned folk who still think it becoming to await appreciation with patience, and believe in the ultimate triumph of the deserving, but they are a small and diminishing number, and most men who covet reputation and reward are fully alive to the advantages of helping themselves. More than this, they have no manner of doubt that they well deserve all they get, and, unlike the great Lord Melbourne, who thought the acquisition of honour all the more agreeable when there was no suspicion of merit to justify it, the ambitious ones of to-day would feel deeply affronted if their statement of account were not accepted with a very large balance to their credit. They never cry "quits"; the prizes, however liberally awarded, are but instalments, and the world remains always their debtor.

It is no fault of the "Albert and Victoria Society," but rather its misfortune, that it has been hitherto unknown in the upper circles of philanthropy. It bears a distinguished name, none the worse because self-bestowed, and it lives in a world of its own, which we now learn is akin to the hospital world. It is one of those beneficent organisations which exist only to do good; but alas! it is not independent of those adverse conditions which affect all benevolent efforts alike. Nothing was more natural than that an institution so useful should be in want, and what could be more appropriate than that it should fall back upon a dinner? Accordingly its friends sat down one evening last week at the Holborn Restaurant, prepared to support its claims with much enthusiasm and a few subscriptions. We remember that a character in one of Bulwer Lytton's best known plays seeks to recommend himself to the lady of his choice by the remarkable assertion that he has not much to spend, but what he has he contrives to ge rid of upon himself. The Albert and Victoria Society is able to go beyond this, and to put forward to a sympathetic public the plea that besides spending all it possesses upon itself, it is enabled to assist other institutions which captious or shortsighted observers might think were only made use of in the process.

"I know," said the Chairman of the evening, "of no association more meritorious." High praise indeed, but mark how it is justified: "The payment of a penny a-week entitles a person to membership, as well as to a letter to a hospital. The Society, therefore, gives valuable aid to the hospitals and teaches workpeople thrift and independence." He must, indeed, be fastidious who does not see in these words the outline of a truly great and laudable enterprise. But as if to emphasise matters, we are told further that a sum of £400 was contributed to hospitals last year, and that 1,239 letters were distributed. Well done Albert and Victoria Society, and well done hospitals, too! We are not told the proportion of in-patients among the 1,239 free and independent sufferers relieved, but nothing can detract from the merit either of the Society which assists or the hospitals which can afford to be assisted on these lines.

No wonder the subscription list at the dinner scarcely rose to the level of the great names which adorned it. What need of large contributions when a little money can be made to go so far? Will the hospitals concerned, who, after all, must know more about it than the Society, kindly say how it is all done? It is distressing to reflect that so many active minds are still busy with the hospital problem when the chief difficulty seems to have been already solved. Agricola.

